# Supplementary material for: What influences home delivery among women who live in urban areas? Analysis of 2014 Ghana Demographic and Health Survey data
Source: PLoS One. 2021 Jan 4;16(1):e0244811. doi: 10.1371/journal.pone.0244811 (PMC7781474; doi:10.1371/journal.pone.0244811)
Supplement: Attachment — Submitted filename: Gahan paper PONE-D-20-23864_reviewer.pdf [file pone.0244811.s002.pdf]

PLOS ONE

What influences home delivery among women who live in urban areas in Ghana? Analysis of 2014 Ghana Demographic and Health Survey data

--Manuscript Draft--

Manuscript Number:	PONE-D-20-23864
Article Type:	Research Article
Full Title:	What influences home delivery among women who live in urban areas in Ghana? Analysis of 2014 Ghana Demographic and Health Survey data
Short Title:	What influences home delivery among women who live in urban areas in Ghana
Corresponding Author:	Eugene Budu, M.PHil<br>University of Cape Coast<br>Cape Coast, GHANA
Keywords:	Home delivery; Maternal mortality; Ghana; Women's health; Public health; Global health
Abstract:	Background<br><br>The present study investigated factors associated with home delivery among urban women in Ghana. Data for this study were obtained from the 2014 Ghana Demographic and Health Survey.<br><br>Materials and Methods<br><br>We used data of 1,441 women aged 15-49 who had ever given birth and were dwelling in urban areas. By the use of STATA version 14.2, we conducted both descriptive and multivariable logistic regression analyses and tested the results at 95% confidence interval.<br><br>Results<br><br>The study revealed that, compared to women who lived in the Northern region, women who lived in the Brong Ahafo region [AOR=0.38, CI=0.17-0.84] were less likely to deliver at home. The likelihood of home delivery was high among women in the poorest wealth quintile [AOR=2.02, CI=1.06-3.86], women who professed other religions [AOR=3.45; 1.53-7.81], and those who had no ANC visits [AOR=7.17; 1.64-31.3]. Conversely, the likelihood of home delivery was low among women who had attained secondary/higher education [AOR=0.30; 0.17-0.53], compared to those with no formal education.<br><br>Conclusion<br><br>The study identified region of residence, wealth quintile, religion, ANC visits, and educational attainment as predictors of home delivery. Therefore, health promotion programs targeted at home delivery need to focus on these factors. We also recommend that a qualitative study be conducted to investigate the factors responsible for the differences in home delivery in terms of region, as the present study could not do so.
Order of Authors:	Bright Opoku Ahinkorah
	Abdul-Aziz Seidu
	Eugene Budu, M.PHil
	Ebenezer Agbaglo
	Francis Appiah
	Collins Adu
	Edward Kwabena Ameyaw

Powered by Editorial Manager® and ProduXion Manager® from Aries Systems Corporation

Additional Information:	
**Question**	**Response**
**Financial Disclosure**<br><br>Enter a financial disclosure statement that describes the sources of funding for the work included in this submission. Review the submission guidelines for detailed requirements. View published research articles from *PLOS ONE* for specific examples.<br><br>This statement is required for submission and **will appear in the published article** if the submission is accepted. Please make sure it is accurate.<br><br>**Unfunded studies**<br>Enter: *The author(s) received no specific funding for this work.*<br><br>**Funded studies**<br>Enter a statement with the following details:<br>• Initials of the authors who received each award<br>• Grant numbers awarded to each author<br>• The full name of each funder<br>• URL of each funder website<br>• Did the sponsors or funders play any role in the study design, data collection and analysis, decision to publish, or preparation of the manuscript?<br>• **NO** - Include this sentence at the end of your statement: *The funders had no role in study design, data collection and analysis, decision to publish, or preparation of the manuscript.*<br>• **YES** - Specify the role(s) played.<br><br>* typeset	The author(s) received no specific funding for this work.
**Competing Interests**<br><br>Use the instructions below to enter a competing interest statement for this submission. On behalf of all authors, disclose any competing interests that could be perceived to bias this work—acknowledging all financial support and any other relevant financial or non-financial competing interests.	The authors have declared that no competing interests exist.

Powered by Editorial Manager® and ProduXion Manager® from Aries Systems Corporation

This statement **will appear in the published article** if the submission is accepted. Please make sure it is accurate. View published research articles from *PLOS ONE* for specific examples.<br><br>**NO authors have competing interests**<br><br>Enter: *The authors have declared that no competing interests exist.*<br><br>**Authors with competing interests**<br><br>Enter competing interest details beginning with this statement:<br><br>*I have read the journal's policy and the authors of this manuscript have the following competing interests: [insert competing interests here]*<br><br>* typeset	
**Ethics Statement**<br><br>Enter an ethics statement for this submission. This statement is required if the study involved:<br><br>• Human participants<br>• Human specimens or tissue<br>• Vertebrate animals or cephalopods<br>• Vertebrate embryos or tissues<br>• Field research<br><br>Write "N/A" if the submission does not require an ethics statement.<br><br>General guidance is provided below. Consult the submission guidelines for detailed instructions. **Make sure that all information entered here is included in the Methods section of the manuscript.**	The survey reported that ethical approval was granted by the Institutional Review Board of ICF International and Ethical Review Committee of Ghana Health Service (Ghana Statistical Service, Ghana Health Service & ICF Macro, 2015). We further obtained permission from the DHS Program for use of this data for the study. Data is available on https:// https://dhsprogram.com/data/dataset

**Format for specific study types**<br><br>**Human Subject Research (involving human participants and/or tissue)**<br>• Give the name of the institutional review board or ethics committee that approved the study<br>• Include the approval number and/or a statement indicating approval of this research<br>• Indicate the form of consent obtained (written/oral) or the reason that consent was not obtained (e.g. the data were analyzed anonymously)<br><br>**Animal Research (involving vertebrate animals, embryos or tissues)**<br>• Provide the name of the Institutional Animal Care and Use Committee (IACUC) or other relevant ethics board that reviewed the study protocol, and indicate whether they approved this research or granted a formal waiver of ethical approval<br>• Include an approval number if one was obtained<br>• If the study involved *non-human primates*, add *additional details* about animal welfare and steps taken to ameliorate suffering<br>• If anesthesia, euthanasia, or any kind of animal sacrifice is part of the study, include briefly which substances and/or methods were applied<br><br>**Field Research**<br><br>Include the following details if this study involves the collection of plant, animal, or other materials from a natural setting:<br>• Field permit number<br>• Name of the institution or relevant body that granted permission	
**Data Availability**<br><br>Authors are required to make all data underlying the findings described fully available, without restriction, and from the time of publication. PLOS allows rare exceptions to address legal and ethical concerns. See the PLOS Data Policy and FAQ for detailed information.	Yes - all data are fully available without restriction

A Data Availability Statement describing where the data can be found is required at submission. Your answers to this question constitute the Data Availability Statement and **will be published in the article**, if accepted.<br><br>**Important:** Stating 'data available on request from the author' is not sufficient. If your data are only available upon request, select 'No' for the first question and explain your exceptional situation in the text box.<br><br>Do the authors confirm that all data underlying the findings described in their manuscript are fully available without restriction?	
**Describe where the data may be found in full sentences. If you are copying our sample text, replace any instances of XXX with the appropriate details.**<br><br>• If the data are **held or will be held in a public repository**, include URLs, accession numbers or DOIs. If this information will only be available after acceptance, indicate this by ticking the box below. For example: *All XXX files are available from the XXX database (accession number(s) XXX, XXX.*).<br>• If the data are all contained **within the manuscript and/or Supporting Information files**, enter the following: *All relevant data are within the manuscript and its Supporting Information files.*<br>• If neither of these applies but you are able to provide **details of access elsewhere**, with or without limitations, please do so. For example:<br><br>*Data cannot be shared publicly because of [XXX]. Data are available from the XXX Institutional Data Access / Ethics Committee (contact via XXX) for researchers who meet the criteria for access to confidential data.*<br><br>*The data underlying the results presented in the study are available from (include the name of the third party*	Data is available on https:// https://dhsprogram.com/data/dataset

and contact information or URL).<br>• This text is appropriate if the data are owned by a third party and authors do not have permission to share the data.<br><br>* typeset	
Additional data availability information:	

Manuscript
Click here to access/download;Manuscript;Final HOME DELIVERY_Vancouver copy.docx

What influences home delivery among women who live in urban areas in Ghana? Analysis of 2014 Ghana Demographic and Health Survey data

Bright Opoku Ahinkorah[1], Abdul-Aziz Seidu[2*], Eugene Budu[2], Ebenezer Agbaglo[3], Francis Appiah[2] Collins Adu[4], Edward Kwabena Ameyaw[1]

[1]The Australian Centre for Public and Population Health Research (ACPPHR), Faculty of Health, University of Technology Sydney, Australia
[2]Department of Population and Health, University of Cape Coast, Cape Coast, Ghana
[3] Department of English, University of Cape Coast, Cape Coast, Ghana
[4]Department of Health Promotion and Disability Study, Kwame Nkrumah University of Science and Technology, Kumasi, Ghana
*Corresponding author
Email addresses:

BOA: brightahinkorah@gmail.com
*AS: abdul-aziz.seidu@stu.ucc.edu.gh
EB: budueugene@gmail.com
EA: ebenezer.agbaglo@stu.ucc.edu.gh
FA: engman477@yahoo.com
CA:collinsadu80@yahoo.com
EKA: edmeyaw19@gmail.com

Abstract

Background

The present study investigated factors associated with home delivery among urban women in Ghana. Data for this study were obtained from the 2014 Ghana Demographic and Health Survey.

Materials and Methods

We used data of 1,441 women aged 15-49 who had ever given birth and were dwelling in urban areas. By the use of STATA version 14.2, we conducted both descriptive and multivariable logistic regression analyses and tested the results at 95% confidence interval.

Results

The study revealed that, compared to women who lived in the Northern region, women who lived in the Brong Ahafo region [AOR=0.38, CI=0.17-0.84] were less likely to deliver at home. The likelihood of home delivery was high among women in the poorest wealth quintile [AOR=2.02, CI=1.06-3.86], women who professed other religions [AOR=3.45; 1.53-7.81], and those who had no ANC visits [AOR=7.17; 1.64-31.3]. Conversely, the likelihood of home delivery was low among women who had attained secondary/higher education [AOR=0.30; 0.17-0.53], compared to those with no formal education.

Conclusion

The study identified region of residence, wealth quintile, religion, ANC visits, and educational attainment as predictors of home delivery. Therefore, health promotion programs targeted at home delivery need to focus on these factors. We also recommend that a qualitative study be conducted to investigate the factors responsible for the differences in home delivery in terms of region, as the present study could not do so.

Keywords: Home delivery; Maternal mortality; Ghana; Women’s health; Public health; Global health

Background

One issue that has always been of public health concern is high maternal mortality. Because of this, the international community has always placed premium on reducing maternal mortality. For example, the then Millennium Development Goals (MDGs) aimed at reducing maternal mortality by 75% by the year 2015 [1,2]. While this aim saw a significant decline in maternal mortality rates in countries all over the world, Ghana could not achieve this target, as maternal mortality reduced only by 45% by the end of 2015 [3]. After the MDGs came the Sustainable Development Goals (SDGs), which also targets improvements in women’s health, including reduction of maternal mortality. Specifically, target 3.1 of the SDGs aims at reducing the global maternal mortality ratio to less than 70 per 100,000 live births by the year 2030 [4,5]. Despite this, the world still struggles with maternal mortality, with sub-Saharan African countries disproportionately affected [5], with 351 per 100,000 live births [6]. In the context of Ghana, as of 2017, the maternal mortality ratio stood at 310 deaths per 100,000 births [7]

A larger percentage of maternal mortalities in Ghana is caused by pregnancy-related issues such as obstetric complications, which result in death during pregnancy, childbirth, or within 42 days after delivery [5]. This implies that maternal mortality can be reduced by ensuring that women

have better maternal healthcare services, including skilled birth delivery [5]. Over the years, the government of Ghana has attempted to improve access to maternal healthcare services. In 2003, the government, for example, introduced the waiver of delivery fees, and by 2005, fees on delivery care were abolished in all the then 10 regions of the country [8]. This was followed by the introduction of the National Health Insurance Scheme (NHIS) in 2005. The NHIS allows all pregnant women under the scheme to have free access to maternal healthcare services, including antenatal care, delivery services, postnatal care, and neonatal care [9]. This program saw a decrease in home delivery from 45% in 2007 to 20% in 2017 [7]. However, there are rural-urban differentials in terms of home delivery, with the prevalence of home delivery in rural areas being 40%, as compared to 10.2% in urban areas [10]. Besides, 9% of women in urban areas are likely to deliver without a skilled provider [11].

While the prevalence of home delivery appears to be relatively low in Ghana, there are still some barriers that obstruct women from using skilled birth attendants, despite the waiver of delivery fees, and this consequently puts women at increased risk of infections and maternal mortality [12, 13]. In view of this, more efforts are needed to significantly reduce maternal mortality in Ghana, if the country is to achieve the objectives of SDG 3.1. To this end, it is needful to reveal factors associated with home delivery, as such factors will provide useful information to interventions aimed at reducing maternal mortality in ~~Ghana~~. In Ghana, a few studies have been conducted in this regard. Studies by Ganle et al. [14] and Boah et al. [15] in Northern Ghana and the Builsa South District of the Upper West region, respectively, identified fewer antenatal care visits, lack of health insurance, living in a male-headed household, being unexposed to media, parity, poor attitude of nurses, lack of transportation, cost of delivery kits, and traditional beliefs and practices as predictors of home delivery. However, these studies focused more on rural areas, ignoring the fact that a considerable number of women in urban areas still use home delivery services. The present study departs from previous ones by assessing the factors associated with home delivery in urban Ghana.

Materials and Methods

Data source

The study used data from the 2014 Demographic and Health Survey (DHS) of Ghana. DHS is a nationwide survey collected every five-year period across low- and middle-income countries. Women's file was used for this study and contains the responses by women aged 15 and 49. The survey targets core maternal and child health indicators such as unintended pregnancy, contraceptive use, skilled birth attendance, immunisation among under-fives, and intimate partner violence. Stratified dual-stage sampling approach was employed and the same questions were posed to all women. Selection of clusters (i.e. enumeration areas [EAs]) is the first step in the sampling process, followed by systematic household sampling within the selected EAs. The second step involves the selection of households from the predefined clusters. For the purpose of this study, only women in the urban areas who had ever given birth and had complete cases on all the variables considered for the study were included (N=1,441). Details of the methodology employed by the GDHS can be found in the final report [16]. We relied on the Strengthening the "Reporting of Observational Studies in Epidemiology" (STROBE) statement in writing the manuscript.

Study variables definition

Outcome variable

The outcome variable employed for this study was "home delivery" which was obtained from the question, "Where did you deliver?" Responses to this question were coded respondent's home, other home, government hospital, government health centre/clinic, government health post/CHPS, other public, private hospital/clinic, maternity homes and others. It was then dichotomised into facility delivery= 0 and home delivery=1, where respondent's home and other home were group as "home delivery" and all the other categories were grouped as "facility delivery."

Explanatory variables

The study considered twelve explanatory variables. These are age, region, religion, ethnicity, educational level, marital status, wealth status, employment, parity, sex of household head, antenatal visits, and decision-making for healthcare. These variables were not determined a priori; instead, they were determined based on parsimony, theoretical relevance, and practical significance with place of delivery [17, 18,19]. The categorization of the variables can be found in Table 1.

Statistical analysis

The statistical software STATA version 14.0 was used to process the data. Both bivariate and multivariate analyses were employed in this study and results were tested at 95% confidence interval. Bivariate analysis was conducted to show the proportion of home deliveries across socio-demographic characteristics with their significance levels and chi-square values ($\chi 2$). Multivariate analysis (binary logistic regression) was further conducted. Only the variables that showed statistical significance in the bivariate analysis were used for the regression analysis. Before the binary logistic regression analysis, we conducted a multicollinearity test of all the statistically significant variables using the variance inflation factor (VIF), and it showed no evidence of collinearity among the explanatory variables (Mean VIF=1.32, Max VIF=1.57, Minimum=1.02**)**. The results were presented as adjusted odds ratios, with their corresponding 95% confidence intervals signifying their level of precision. Statistical significance was declared at $p<0.05$. Sample weight was applied and the survey command (svy) was used to account for the complex sampling design of the survey.

Ethical Approval

The survey reported that ethical approval was granted by the Institutional Review Board of ICF International and Ethical Review Committee of Ghana Health Service (Ghana Statistical Service, Ghana Health Service & ICF Macro, 2015). We further obtained permission from the DHS Program for use of this data for the study. Data is available on https:// https://dhsprogram.com/data/dataset

Results

Distribution of the prevalence of home deliveries among women in the urban areas

Table 1 presents results on the distribution of the prevalence of home deliveries among women in the urban areas of Ghana across socio-demographic characteristics. The prevalence of home deliveries in the country was 7.9%, with variations across the various socio-demographic characteristics of the respondents. The results of the chi-square test showed that region, ethnicity,

educational level, wealth index, parity, religion, and ANC visits had significant associations with home delivery ($p<0.001$).

*****Table 1*****

Predictors of home delivery among women in the urban areas of Ghana

Table 2 shows results on the predictors of home delivery among women in the urban areas of Ghana**.** Compared to women who lived in the Northern region, women who lived in the Brong Ahafo region [AOR=0.38, CI=0.17-0.84] were less likely to deliver at home. Women with poorest wealth quintile were more likely to deliver at home, compared to those with middle wealth quintile [AOR=2.02, CI=1.06-3.86]. The likelihood of home delivery was high among women who professed other religions, compared to Christians [AOR=3.45; 1.53-7.81]. Home delivery was found to be high among women who had no ANC visits, compared to those who had at least one ANC visit [AOR=7.17; 1.64-31.3]. Conversely, the likelihood of home delivery was low among women who had attained secondary/higher education [AOR=0.30; 0.17-0.53], compared to those with no formal education.

*****Table 2*****

Discussion

Home delivery presents an array of negative health complications to the mother and the child; hence, ensuring health facility delivery has the potential to avert such risks [5]. Therefore, the primary focus of our study was to assess the factors associated with home delivery in urban Ghana. Our study revealed that, compared to all the regions, women who lived in the Northern region were more likely to deliver at home. Debatably, health facilities in Ghana are not equally distributed, and disfavours some regions. Typically, the northern part of the country is less endowed with health facilities [20, 21].

Several studies have further remarked that accessibility and availability of quality maternal health services influence the use of an institution for delivery [22, 23,24,25,26]. Shahabuddin et al. [27] similarly noted that young women from mountain region Nepal were less likely to choose institutional delivery, compared with women in the Terai region. This implies that, without equitable distribution of health facilities and elimination of accessibility barriers including provision of efficient and effective referral services, health facility delivery will be cumbersome for most women in Ghana who are in the disadvantaged regions.

We found that women with poorest wealth quintile had higher odds to deliver at home, compared to those with middle wealth quintile. Our results is in line with previous studies in other low- and middle-income countries such as Nepal [27], Malawi [28] and Guinea-Bissau [29]. It is increasingly known that wealthier women are more likely to deliver in healthcare facilities than their poorer counterparts [30, 31, 32]. What might have caused this disparity in institutional delivery among the rich and the poor could possibly be attributed to financial stands. Poor women might be challenged financially when there is the need to deliver in a health facility, including the cost of transport and buying other items needed for delivery.

Our study also revealed that those who professed other religions had higher propensity to home delivery, compared to Christians. Religious affiliation was responsible for the disparity in institutional delivery in other studies. For instance, in Nepal, Shahabuddin et al. [27] observed that young Muslim women were 1.82 times more likely to deliver at an institution, compared with young Hindu women. We must acknowledge that religious affiliation comes with specific beliefs

and practices, which may influence women’s general practices including opting health facility delivery [14,15]. Our study fails to unravel why women from other religious backgrounds were more likely to deliver at home, compared to Christians. We, therefore, suggest that a qualitative study be done between religion and place of delivery to understand the phenomenon better.

It is common knowledge that ANC utilisation has a spilt over effect on mothers’ choice of place of delivery, whereby women who utilize ANC mostly prefer institutional delivery supervised by health professionals [33,34]. As such, it was not surprising that home delivery was found to be high among women who had no ANC visits, as compared to those who had at least one ANC visit in our current study. A systematic review in assessing factors influencing utilisation of maternal health services by adolescent mothers in low- and middle-income countries concluded that the use of antenatal care predicted use of skilled birth attendance, and use of both predicted use of postnatal care [35]. Practically, it is suggestive that sufficient ANC utilisation is likely to increase pregnant women's awareness of possible complications and safe delivery practices, which consequently urges them to deliver in a health facility [36, 37, 38, 39]. It has also been argued that women who visit health facilities for healthcare services including ANC check-ups might be exposed to counselling and guidance from health professionals [40]. Both scenarios enlighten them about the dangers associated with home deliveries. Theoretically, following the proposition raised by the protection motivation theory (PMT), women, having been exposed to vital information during ANC, will opt for delivering at a health facility to serve as a protection against unforeseen predicaments associated with home delivery [41, 42, 43].

Finally, we noted that the probability to deliver at home was low among women who had attained secondary/higher level of education, compared to those with no formal education. Similarly, studies in Malawi observed that women who had no formal education were about four-fold probable to deliver at home, compared to women who had attained secondary or higher level [28]. In a related study, Yaya, Bishwajit, and Gunawardena [29] also found that, among residents in urban areas of Guinea-Bissau, compared those who had no education, those who had primary and secondary/higher level of education were 2.2 and 3.3 times more likely to deliver at a health facility. Level of education has been a determinant to home delivery in Zambia as well, and it was known that women who had four years of schooling or less were 63 percent more likely to deliver at home than a health facility, compared to those who had at least five years of schooling. A recent study also found that having education increases the likelihood that a woman would decide to deliver her baby in a hospital or maternity home than at home or in other places [44]. Oyedele [44] further explained that education increases individuals’ awareness about health holistically and exposes them to benefits associated with complication prevention. Therefore, we can argue that the educated Ghanaians would be compelled to live a sanitary and hygienic life including opting institutional delivery.

Strengths and limitations

Among the strengths of the study is the fact that it depended on a nationally representative data set. Also, the study adopted probability method in selecting survey respondents and used sound analytical procedure which enhances the robustness of the results. However, our results should be interpreted with caution since causality cannot be established.

Conclusion

The major determinants of home delivery among urban residents observed in our study were region of residence, wealth quintile, religion of affiliation, ANC visits, and educational attainment. What might have compelled Northern residents to be inclined to home delivery calls for health education emphasising health facility delivery while prioritising health facility delivery on policy initiatives. Women should be empowered to utilise ANC, using sufficient counselling, guidance, and mass sensitisation through various mass media platforms. Disparity in health facility delivery masked by religion of affiliation calls for a qualitative study to interrogate the phenomenon.

Abbreviations

ANC: Antenatal Care; AOR: Adjusted Odds Ratio; CI: Confidence Interval; DHS: Demographic and Health Surveys; MMR: Maternal Mortality Ratio; WHO: World Health Organization; SDG: Sustainable Development Goal; SSA: sub-Saharan Africa; LMICs: Low and Middle-income countries ; PNG: Papua New Guinea

Acknowledgements

We acknowledge Measure DHS for providing us with the data.

Competing interests

The authors declare that they have no competing interests.

References

1. Brizuela V & Tunçalp Özge. Global initiatives in maternal and newborn health. *Obstet Med.* 2017:10:21–25.

2. Alkema L, Chou D, Hogan D, *et al*. Global, regional, and national levels and trends in maternal mortality between 1990 and 2015, with scenario-based projections to 2030: A systematic analysis by the UN maternal mortality estimation Inter-Agency group. *The Lancet.* 2016:387:462–74.

3. Bhowmik J, Biswas RK & Woldegiorgis M. Antenatal care and skilled birth attendance in Bangladesh are influenced by female education and family affordability: BDHS 2014. *Public Health.* 2019:170:113-21.

4. Badiuzzaman M., Murshed S. M. & Rieger, M. Improving maternal health care in a post conflict setting: Evidence from Chittagong Hill tracts of Bangladesh. *J Dev Stud.* 2018:36:1-17.

5.World Health Organization. *Primary health care on the road to universal coverage: 2019 global monitoring report*. WHO.2019.

6. Ahmed I., Ali S. M., Amenga-Etego S., Ariff S., Bahl R., Baqui A. H & Biemba, G. Population-based rates, timing and causes of maternal deaths, stillbirths, and neonatal deaths in south Asia and sub-Saharan Africa: a multi-country prospective cohort study. *The Lancet Global Health*.2018:6(12):1297-1308.

7. Ghana Statistical Service (GSS), Ghana Health Service (GHS), and ICF International. *Ghana Maternal Health Survey 2017: Key Findings.* Rockville, Maryland, USA: GSS, GHS, and ICF 2018.

8. Koduah A, Van Dijk H & Agyepong IA. The role of policy actors and contextual factors in policy agenda setting and for- mulation: Maternal fee exemption policies in Ghana over four and a half decades. *Health Res Pol Syst*. 2015:13: 27.
9. Wang W, Temsah G & Mallick L. The impact of health insur- ance on maternal health care utilization: evidence from Ghana, Indonesia and Rwanda. *Health Pol Plann*. 2017: 32: 366–375.
10. Dankwah E., Zeng W., Feng C., Kirychuck S., & Farag M. The social determinants of health facility delivery in Ghana. Reproductive Health.2019: 16(101)
11. Ghana Statistical Service, Ghana Health Service, ICF International. Ghana Demographic and Health Survey, 2014. Rockville, Maryland, USA; 2015.
12. Abebe F, Berhane Y & Girma B. Factors associated with home delivery in Bahirdar, Ethiopia: A case control study. *BMC Res Notes*. 2012: 5: 653. https://doi.org/10.1186/1756-0500-5-653 PMID: 23176369 .
13.Envuladu EA, Agbo HA, Lassa S, Kigbu JH & Zoakah AI. Factors determining the choice of a place of delivery among pregnant women in Russia village of Jos North, Nigeria: achieving the MDGs 4 and 5. *Int J Med Biomed Res*. 2013: 2: 23–27.
14. Ganle, K. K., Mahama, M. S., Maya, E., Manu, A., Torpey, K., & Adanu, R. Understanding factors influencing home delivery in the context of user- fee abolition in Northern Ghana: Evidence from 2014 DHS. *International Journal of Health Planning Management*. 2019: 1–17.
15. Boah M, Adampah T, Jin B, Wan S, Mahama AB, Hyzam D & Akiti, C. “I couldn’t buy the items so I didn’t go to deliver at the health facility” Home delivery among rural women in northern Ghana: A mixed-method analysis. *PLoSONE*. 2020:15(3):0230341. https://doi.org/10.1371/journal.pone.0230341
16. Ghana Statistical Service (GSS), Ghana Health Service (GHS), and ICF International. Ghana Demographic and Health Survey 2014. Rockville: GSS, GHS, and ICF International; 2015.
17. De Allegri M., Riddle V., Louis V., Sarker M., Trendrebéogo J. & Yé, M. Determinants of utilization of maternal care services after the reduction of user fee: A case study from rural Burkina Faso. *Health Policy*.2011: 99: 210-218.
18. Spangler, S. A. & Bloom, S. S. Use of biomedical obstetric care in rural Tanzania: The role of social and material inequality. *Social Science and Medicine*. 2010:71(4):760-768.
19. Adanu, R. M. Utilisation of obstetric services in Ghana between 1999 to 2003. *African Journal of Reproductive Health*. 2010:14(3):153-158.
20. Akum, F. A. A qualitative study on factors contributing to low institutional child delivery rates in Northern Ghana: The case of Bawku Municipality. *J Community Med Health Educ*. 2013: 3(6): 1-9.
21. Ghana Statistical Service (GSS), Ghana Health Service (GHS), and ICF Macro. Ghana Demographic and Health Survey 2008. Accra, Ghana: GSS, GHS, and ICF Macro. 2009.
22. Duysburgh E, Zhang WH, Ye M, *et al*. Quality of antenatal and childbirth care in selected rural health facilities in Burkina Faso, Ghana and Tanzania: similar finding. *Trop Med Int Health*. 2013:18:534-47.
23. Karkee R., Binns C. W. & Lee A. H. Determinants of facility delivery after implementation of safer mother programme in Nepal: A prospective cohort study. *BMC Pregnancy Childbirth.* 2013:13:193.
24. Gebrehiwot T, San Sebastian M, Edin K, *et al*. Health workers' perceptions of facilitators of and barriers to institutional delivery in Tigray, Northern Ethiopia. *BMC Pregnancy Childbirth*.2014:14:1-10.

25. Morrison J, Thapa R, Basnet M, *et al*. Exploring the first delay: A qualitative study of home deliveries in Makwanpur district Nepal. *BMC Pregnancy Childbirth.* 2014:14:89.
26. De Brouwere, V., Richard, F. & Witter, S. Access to maternal and perinatal health services: lessons from successful and less successful examples of improving access to safe delivery and care of the newborn. *Trop Med Int Health* 2015;15:901–9.
27. Shahabuddin A. S. M., De Brouwere V., Adhikari R., Delamou A., Bardaj A. & Delvaux, T. Determinants of institutional delivery among young married women in Nepal: Evidence from the Nepal Demographic and Health Survey. *BMJ Open*. 2017:7:012446. doi:10.1136/bmjopen-2016-012446.
28. Palamuleni, M. Determinants of non-institutional deliveries in Malawi. *Malawi Medical Journal*. 2011: 23(4): 104-108.
29. Yaya S., Bishwajit G. & Gunawardena N. Socioeconomic factors associated with choice of delivery place among mothers: A population-based cross-sectional study in Guinea-Bissau. *BMJ Glob Health*. 2019: 4:001341. doi:10.1136/ bmjgh-2018-001341
30. Wagle R. R., Sabroe S. & Nielsen B. B. Socioeconomic and physical distance to the maternity hospital as predictors for place of delivery: An observation study from Nepal. *BMC Pregnancy Childbirth*. 2004: 4.
31. Agha S. & Carton T. W. Determinants of institutional delivery in rural Jhang, Pakistan. *Int J Equity Health.* 2011:10.
32. Kitui J., Lewis S. & Davey G. Factors influencing place of delivery for women in Kenya: An analysis of the Kenya demographic and Health survey, 2008/2009. *BMC Pregnancy Childbirth.* 2013: 13.
33. Dahiru T. & Oche O. M. Determinants of antenatal care, institutional delivery and postnatal care services utilization in Nigeria. *Pan African Medical Journal*. 2015: 21(321):2-9. doi:10.11604/pamj.2015.21.321.6527
34. Pervin J, Moran A, Rahman M, Abdur Razzaque, Sibley L, Streatfield PK et al. Association of antenatal care with facility delivery and perinatal survival- a population-based study in Bangladesh. *BMC Pregnancy and Childbirth*. 2012:12:111.
35. Banke-Thomas O. E., Banke-Thomas A. O. & Ameh C. A. Factors influencing utilisation of maternal health services by adolescent mothers in Low-and middle-income countries: A systematic review. *BMC Pregnancy and Childbirth*. 2017:17(65):1-14. DOI 10.1186/s12884-017-1246-3
36. Rahman M. & Mosiur R. Deliveries among adolescent mothers in rural Bangladesh: Who provides assistance? *World Health Popul*. 2009:11:5–14. http:// ovidsp. ovid. com/ ovidweb. cgi? T= JS& PAGE=reference& D= med5& NEWS= N& AN= 20057269
37. Kamal S. M. Preference for institutional delivery and caesarean sections in Bangladesh. *J Health Popul Nutr*. 2013: 31:96-109.
38. Feyissa T. R. & Genemo G. A. Determinants of institutional delivery among childbearing age women in western Ethiopia, 2013: Unmatched case control study. *PLoSOne.* 2014:9:97194–7.
39. Berhan Y. & Berhan A. Antenatal care as a means of increasing birth in the health facility and reducing maternal mortality: A systematic review. *Ethiop J Health Sci.* 2014: 24: 93-104.
40. Ghimire U. The effect of maternal health service utilization in early initiation of breastfeeding among Nepalese mothers. *International Breastfeeding Journal*. 2019:14(33):2-8. https://doi.org/10.1186/s13006-019-0228-7.
41. Bandura A. Self-efficacy: Toward a unifying theory of behavioural change. *Psychological Review*. 1997:84:191-215.

42. Floyd D. L., Prentice-Dunn S. & Rogers R. W. A meta-analysis of research on protection motivation theory. *Journal of Applied Social Psychology*. 2000: 30: 407-429.
43. MacDonell K., Chen X., Yan Y., Li F., Gong J., Sun H., Li X. & Stanton B. A protection motivation theory-based scale for tobacco research among Chinese youth. *Journal of Addiction Research and Therapy*. 2013:4(3): 2-7.
44. Oyedele, O. Determinants of maternal healthcare utilization in Nigeria. *African Research Review*.2017: 11(2):283-293.

Table 1: Weighted distribution of the prevalence of home deliveries among women in the urban areas of Ghana across socio-demographic characteristics (N=1,441)

Variable	Frequency (N)	Percentage (%)	Home delivery (%)	χ^2 (p-value)
**Age**				0.57 (0.75)
15-24	174	12.1	15.5	
25-34	787	55.6	53.5	
35 years or more	481	33.4	31.0	
**Region**				101.64 (p<0.001)
Western	109	7.5	4.9	
Central	107	7.4	11.3	
Greater Accra	437	30.4	5.6	
Volta	80	5.6	12.0	
Eastern	118	8.2	7.0	
Ashanti	333	23.1	7.8	
Brong Ahafo	116	8.1	9.1	
Northern	94	6.5	33.8	
Upper East	31	2.1	7.0	
Upper West	15	1.0	1.4	
**Occupation**				0.04 (0.840)
Working	238	16.5	18.3	
Not working	1203	83.5	81.7	

**Ethnicity**				16.32 (p<0.001)
Akan	748	51.9	29.6	
Ga/Dangme	131	9.1	4.2	
Mole Dagbani	235	16.1	39.4	
Other	359	22.8	26.8	
**Educational level**				130.98 (p<0.001)
No education	222	15.4	54.9	
Primary	212	14.7	23.2	
Secondary/Higher	1,007	69.9	21.8	
**Wealth index**				153.31 (p<0.001)
Poorest	67	4.7	26.1	
Poorer	80	5.5	23.2	
Middle	232	16.1	29.6	
Richer	443	30.7	15.5	
Richest	619	43.0	5.6	
**Parity**				30.30 (p<0.001)
One birth	292	20.3	8.5	
Two births	364	25.2	17.6	
Three births	299	20.8	21.1	
Four or more births	486	33.7	52.8	
**Religion**				88.46 (p<0.001)
Christianity	1151	79.9	45.8	
Islam	247	17.1	40.1	
Other	43	3.0	8.5	
**Marital status**				0.72 (0.397)
Married	1084	75.2	75.3	
Cohabiting	357	24.8	24.7	
**Sex of household head**				0.68 (0.411)
Male	1141	79.1	77.5	
Female	300	20.9	22.5	
**ANC visits**				50.48 (p<0.001)
No ANC visits	16	1.1	7.0	
Had ANC visit	1425	98.9	93.0	
**Healthcare decision-making**				0.18 (0.675)
Not alone	1035	71.8	76.1	
Respondent alone	406	28.2	23.9	
National (Total)	1,441	100	7.9	

Source: Computed from 2014 GDHS

Table 2: Logistic regression analysis on predictors of home delivery among women in the urban areas of Ghana

Variable		95% CI	
	**AOR**	**Lower Bound**	**Upper Bound**
**Region**			
Western	0.63	0.20	2.01
Central	0.74	0.29	1.87
Greater Accra	0.42	0.14	1.28
Volta	0.80	0.34	1.90
Eastern	0.54	0.21	1.38
Ashanti	0.59	0.23	1.49
Brong Ahafo	0.38^{*}	0.17	0.84
Northern	**Ref**	**Ref**	**Ref**
Upper East	0.26^{**}	0.11	0.60
Upper West	0.15^{**}	0.04	0.62
**Ethnicity**			
Akan	1.39	0.69	2.79
Ga/Dangme	1.71	0.55	3.36
Mole Dagbani	**Ref**	**Ref**	**Ref**
Other	0.86	0.48	1.56
**Education**			
No education	**Ref**	**Ref**	**Ref**
Primary	0.80	0.46	1.40
Secondary/Higher	0.30^{***}	0.17	0.53
**Wealth**			
Poorest	2.02^{*}	1.06	3.86
Poorer	1.84	0.99	3.41
Middle	**Ref**	**Ref**	**Ref**
Richer	0.38^{***}	0.21	0.68
Richest	0.18^{***}	0.08	0.42
**Parity**			
One birth	0.52	0.26	1.02
Two births	0.96	0.54	1.69
Three births	1.36	0.80	2.31
Four or more births	**Ref**	**Ref**	**Ref**
**Religion**			
Christianity	**Ref**	**Ref**	**Ref**
Islam	0.97	0.53	1.75
Other	3.45^{***}	1.53	7.81
**ANC visits**			
No ANC visits	7.17^{**}	1.64	31.3
Had ANC visit	**Ref**	**Ref**	**Ref**
N	1441		
Pseudo R^2	0.259		

Exponentiated coefficients; 95% confidence intervals in brackets, [*] $p < 0.05$, [**] $p < 0.01$, [***] $p < 0.001$, aOR=adjusted Odds Ratios
