# Supplementary material for: What influences home delivery among women who live in urban areas? Analysis of 2014 Ghana Demographic and Health Survey data
Source: PLoS One. 2021 Jan 4;16(1):e0244811. doi: 10.1371/journal.pone.0244811 (PMC7781474; doi:10.1371/journal.pone.0244811)
Supplement: Attachment — Submitted filename: Response to Editors comments.docx [file pone.0244811.s003.docx]

**AUTHORS’ RESPONSE TO EDITOR’S COMMENTS**

**Title:** What influences home delivery among women who live in urban areas in Ghana? Analysis of 2014 Ghana Demographic and Health Survey data

**Dear Editor,**

Thank you for your email dated 10 December 2020 enclosing the Editor’s comments. We convey our gratitude to you for the comment that has led to the massive improvement of our paper entitled *“***What influences home delivery among women who live in urban areas in Ghana? Analysis of 2014 Ghana Demographic and Health Survey data**”. We have now proofread the paper to correct grammar and spelling errors. All the changes are in tack changes in the revised manuscript. We believe the manuscript has improved substantively and will be published in your reputable journal.

**Version 2:** PONE-D-20-23864R2

**Date: 11/12/2020**

Editor’s comment

Dear Dr. Budu,

Thank you for submitting your manuscript to PLOS ONE. After careful consideration, we feel that it has merit but does not fully meet PLOS ONE’s publication criteria as it currently stands. Therefore, we invite you to submit a revised version of the manuscript that addresses the points raised during the review process.

Carefully proof your manuscript to correct grammar or spelling errors.

Response: We have now proofread the paper to correct grammar and spelling errors

Thank you

Sincerely

Eugene Budu
